# Supplementary material for: Facile construction of fully sp2-carbon conjugated two-dimensional covalent organic frameworks containing benzobisthiazole units
Source: Nat Commun. 2022 Jan 10;13:100. doi: 10.1038/s41467-021-27573-1 (PMC8748616; doi:10.1038/s41467-021-27573-1)
Supplement: Supplementary file 1 — Supplementary Information [file 41467_2021_27573_MOESM1_ESM.pdf]

Facile construction of fully sp^2-carbon conjugated two-dimensional covalent organic frameworks containing benzobisthiazole Units

Yuancheng Wang, Wenbo Hao, Hui Liu, Renzeng Chen, Qingyan Pan, Zhibo Li and Yingjie Zhao**

College of Polymer Science and Engineering, Qingdao University of Science and Technology, Qingdao 266042, China

[*] Corresponding Authors: zbli@qust.edu.cn; yz@qust.edu.cn

Supporting Information

Table of Contents

1. Supporting Methods

1.1 General materials and methods S2

1.2 Synthesis procedure S4

2. Supporting Figures and Legends

Supplementary Figure 1: FT-IR spectra S4

Supplementary Figure 2: FT-IR spectra S5

Supplementary Figure 3: Thermogravimetric analysis profiles S5

Supplementary Figure 4: SEM and TEM images S6

Supplementary Table 1: The HER and AQYs of BTH-1, 2, 3 S7

Supplementary Figure 5: Pore size and pore size distribution profiles S7

Supplementary Figure 6: Solid-state absorption spectrum S8

Supplementary Figure 7: Cycling test of **BTH-3** under irradiation S8

Supplementary Figure 8-9: NMR spectra S9

Supplementary Figure 10: HR-MS spectrum S10

1. Supporting Methods

1.1 General materials and methods

Unless otherwise specified, all reactions were performed in dried glassware under ambient atmosphere. All other reagents were purchased commercially and used without further purification. Organic solvents including ethanol, dichloromethane (DCM), petroleum ether, tris(2-hydroxy ethyl)amine (TEOA), ascorbic acid, tetrahydrofuran, $AcONH_4$, DBU, Cs_2CO_3 and NaOH were purchased from Adamas; acetic acid and 1,4-dioxane were purchased from Alfa Aesar; dilute hydrochloric acid was purchased from Yantai Far East Fine Chemical Co., Ltd. All aqueous solutions were prepared with Milli-Q water.

^{1}H and ^{13}C NMR spectra were performed on 400 MHz spectrometers (Bruker AVANCE NEO 400 Ascend) in the indicated solvents at room temperature. High-resolution solid-state NMR spectra were recorded on Agilent NMR Spectrometer (60054-ASC) using a standard CP pulse sequence probe with 4 mm (outside diameter) zirconia rotors.

Scanning electron microscope (SEM) images were collected using scanning electron microscope (JEOL, JSM-7500F) at an accelerating voltage of 5.0 kV. Transmission electron microscope (TEM) was performed on a JEM-2100 electron microscope with an accelerating voltage of 200 kV.

TGA was carried out on an American TA-Q20 in nitrogen atmosphere using a 10 °C/min ramp without equilibration delay.

The Solid-state UV-Vis absorbance was measured by UV spectrophotometer (HITACHI, U-3900).

Powder X-ray diffraction (PXRD) patterns were obtained on a PANalytical Empyrean X-ray diffractometer with Cu Kα line focused radiation at 40 kV and 40 mA from 2θ = 1.5° up to 40° with 0.02° increment by Bragg-Brentano. The powdered sample was added to the glass and compacted for measurement.

N_2 adsorption isotherms were measured up to 1 bar at 77 K using a Micrometrics ASAP 2460 surface area analyzer. Prior to measurements, samples (ca. 100 mg) were degassed for over 12 h at 120 °C. UHP grade N_2 and He were used throughout the adsorption experiments. Oil-free vacuum pumps and oil-free pressure regulators were used for measurements to prevent contamination of the samples during the degassing process and isotherm measurement.

Photocurrent measurements were performed on the CHI 760E electrochemical system (Shanghai, China), using indium fluorine doped oxide coated glass slides as the working electrode, Ag/AgCl as the reference electrode, and platinum wire as the counter electrode in a three-electrode system. The mixed binder solution is made up of 75 microliters (polytetrafluoroethylene emulsion) and ethanol (675 microliters). The solution was mixed with the COF (5 mg), the COF sample was dispersed by ultrasound, and the solution (50 μl) was dropped onto the FTO glass working electrode (active area 1 cm^2). The samples were dried at 50°C for 30 minutes. N_2 was used to purify the three electrode systems for 5min before measurement. Measurements were performed in a 0.2 M Na_2SO_4 solution under a bias of 0.2 V and the back of the FTO working electrode was illuminated with a solar simulator (1 Sun, ABA grade).

The photocatalytic hydrogen generation experiments of the photocatalysts were performed on a set of photocatalytic equipment (Labsolar-6A). First of all, the as-synthesized COFs of 5 mg was ultrasonically dispersed in 0.1 M ascorbic acid to form a well-dispersed polymer suspension. 20 μl (8 wt% H_2PtCl_6) was then added to the reaction system. After the photocatalytic system was degassed to remove the dissolved air, the suspension was irradiated with a 300 W Xe lamp under vacuum with stirring. Circulating cooling water was used to keep the photocatalytic reaction temperature at 5 °C. A 420 nm cut-off filter was used to filter off ultraviolet light to obtain the simulated visible light irradiation ($\lambda > 420$ nm). The produced hydrogen was measured online by using a gas chromatograph.

Structural modelings of **BTH-1, 2, 3** were performed using the Accelrys Materials Studio software package. The space groups were obtained from the Reticular Chemistry

Structure Resource. P6/M was chosen for AA stacking pattern. The theoretical models were then optimized by the Forcite module. Pawley refinements of the PXRD patterns were done in the Reflex module. The integrated intensities were extracted using Pseudo-Voigt profile. The unit cell parameters a, b, c, FWHM parameters U, V, W, profile parameters NA, NB, and zero point were refined.

1.2 Synthesis procedure

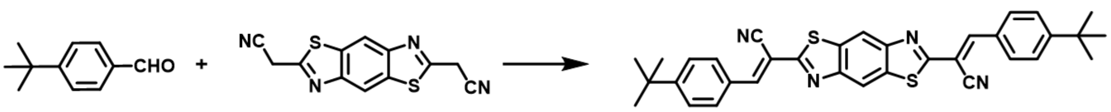

Supplementary Figure 1. Synthetic routes of the model compound.

Synthesis of Model compound

A thick-walled pressure bottle was charged with **BTH-2CN** (100 mg, 0.37 mmol), 4-tert-Butylbenzaldehyde (240 mg, 1.48 mmol), $AcONH_4$ (57 mg, 0.74 mmol) and tetrahydrofuran (20 mL). After being degassed by freeze-pump-thaw technique for three times, the mixture was heated to 100 °C and stirred for 72 hours. After cooling to room temperature, the solvent was removed under vacuum. The obtained solid was washed with water and ethanol to give a crude product. Then, the pure product was recrystalled from dichloromethane and ethanol to give a yellow powder (176.3 mg, yield 85.3%). ^{1}H NMR (400 MHz, $CDCl_3$, δ): 8.57 (s, 2H), 8.26 (s, 2H), 8.02 (d, J = 8.0 Hz, 4H), 7.56 (d, J = 8.0 Hz, 4H), 1.38 (s, 18H). ^{13}C NMR (100 MHz, $CDCl_3$, δ): 164.7, 156.8, 151.9, 147.8, 134.7, 130.6, 129.6, 126.4, 116.5, 115.9, 104.5, 35.3, 31.0. HR-MS (MALDI-TOF-MS) m/z: $[M+H]^+$ calcd. for $C_{34}H_{31}N_4S_2$ 559.1985; found 559.1987.

2. Figures and Legends

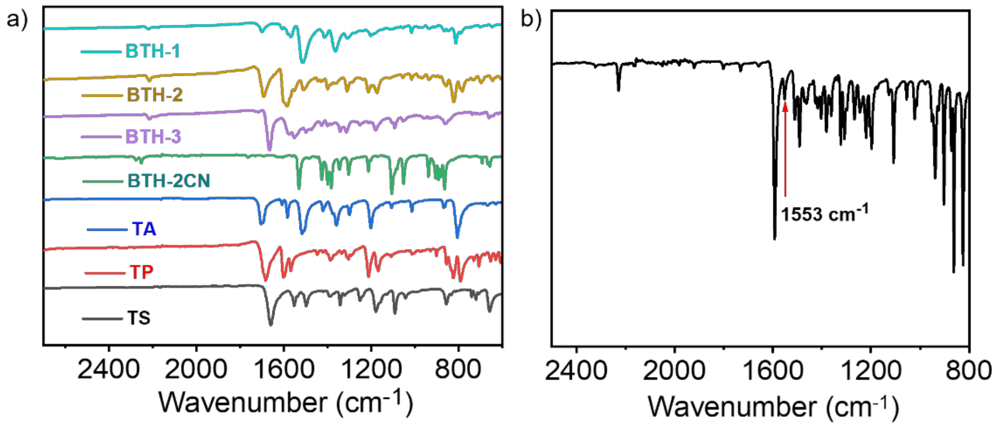

Supplementary Figure 2. (a) The FT-IR spectra of **BTH-1, 2, 3** and the monomers; (b) FT-IR spectra of the model compound.

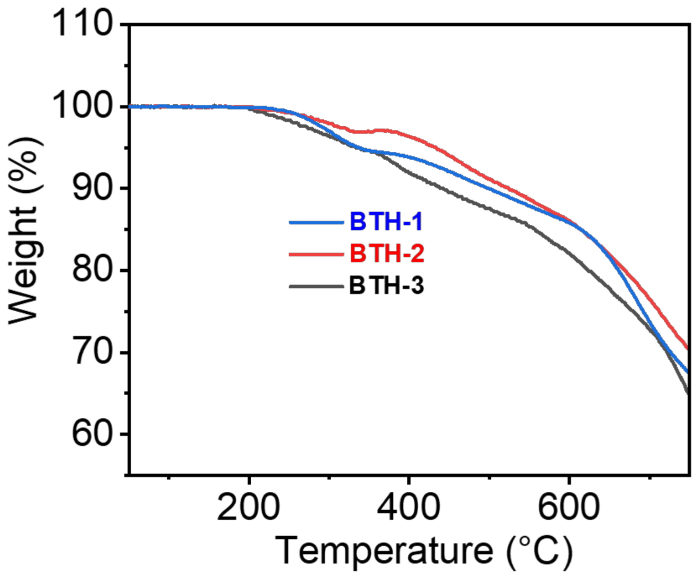

Supplementary Figure 3. The thermogravimetric analysis profiles of **BTH-1, 2, 3**.

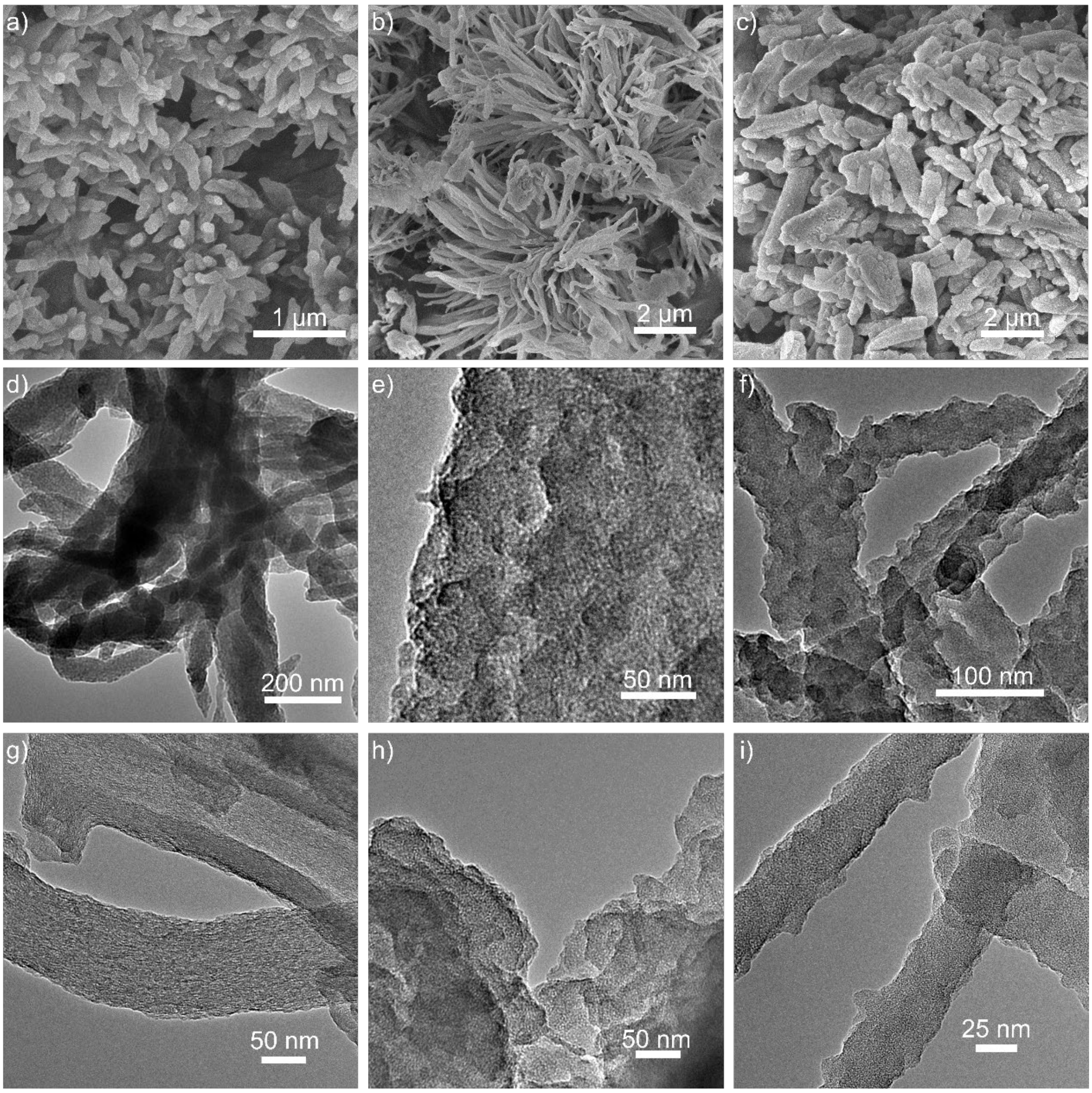

Supplementary Figure 4. SEM images of **BTH-1** (a), **BTH-2** (b) and **BTH-3** (c) and TEM images of **BTH-1** (d, g), **BTH-2** (e, h) and **BTH-3** (f, i).

Supplementary Table 1. The HER and AQYs of BTH-1, 2, 3.

	**BTH-1**	**BTH-2**	**BTH-3**
**HER**	10.5 mmol $h^{-1}g^{-1}$	1.2 mmol $h^{-1}g^{-1}$	15.1 mmol $h^{-1}g^{-1}$
**AQY**(420 nm)	1.569%	0.2%	0.792%
**AQY**(450 nm)	1.089%	0.124%	0.676%
**AQY**(500 nm)	1.925%	0.241%	1.256%
**AQY**(550nm)	0.75 %	0.046%	0.883%
**AQY**(600 nm)			0.735%

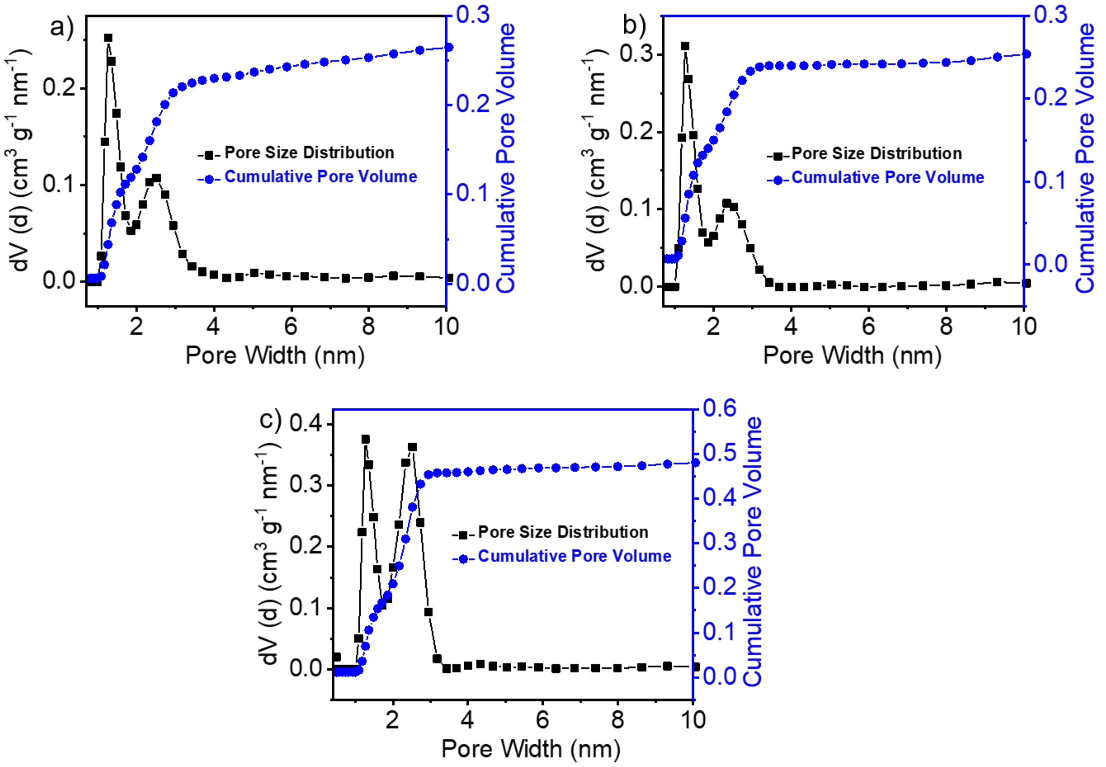

Supplementary Figure 5. Pore size distribution (black) and cumulative pore volume (blue) profiles of **BTH-1** (a), **BTH-2** (b) and **BTH-3** (c).

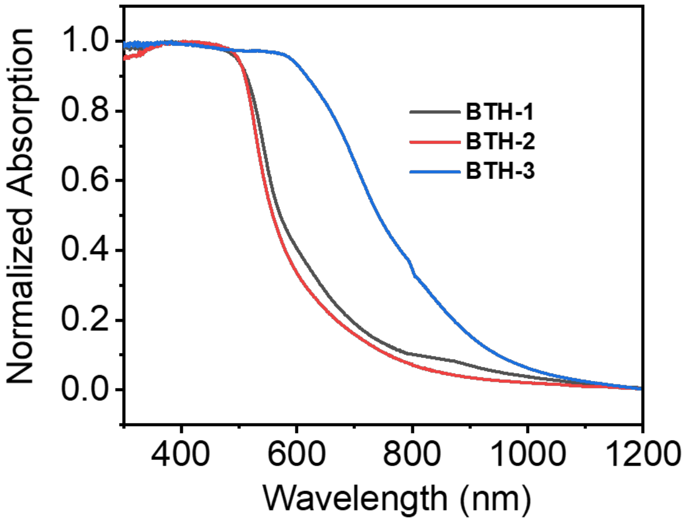

Supplementary Figure 6. The solid-state absorption spectrum of **BTH-1, 2, 3**.

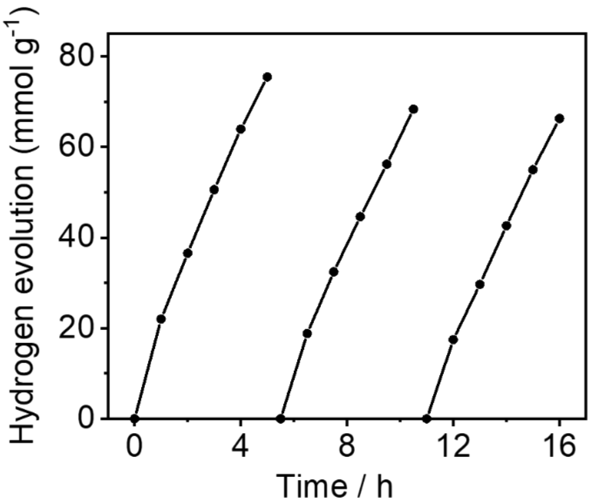

Supplementary Figure 7. Cycling test of **BTH-3** under irradiation.

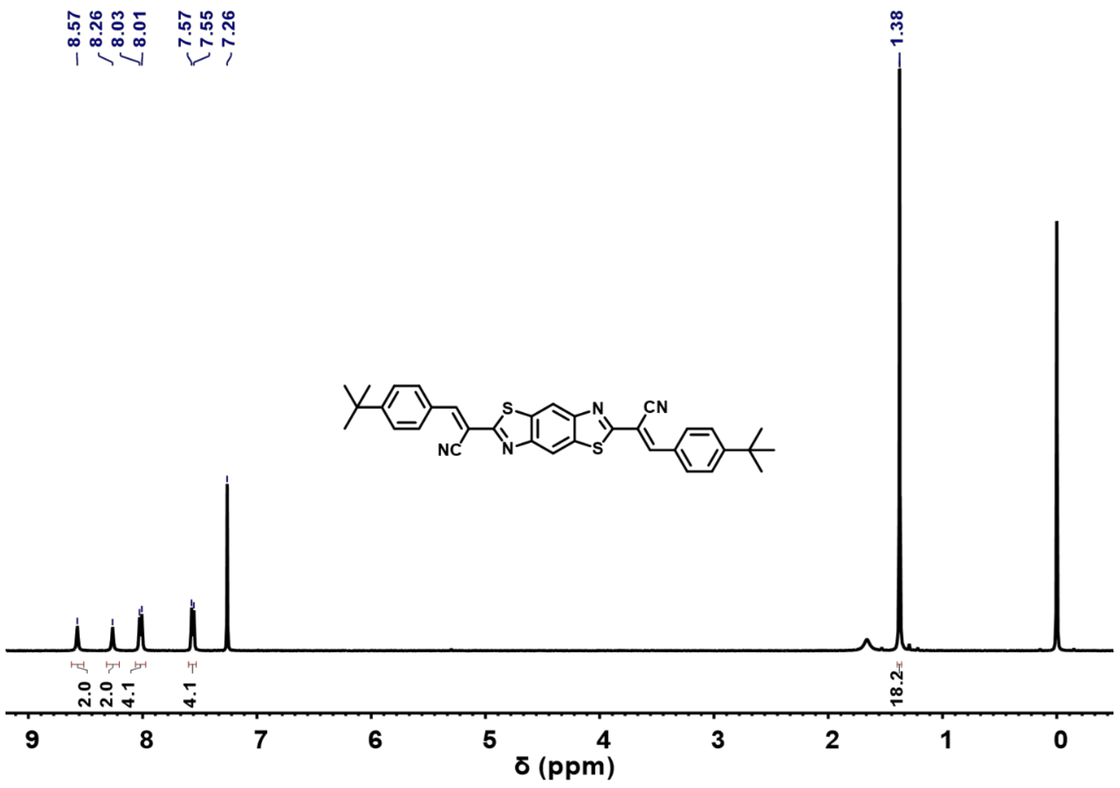

Supplementary Figure 8. ^{1}H NMR spectrum of model compound.

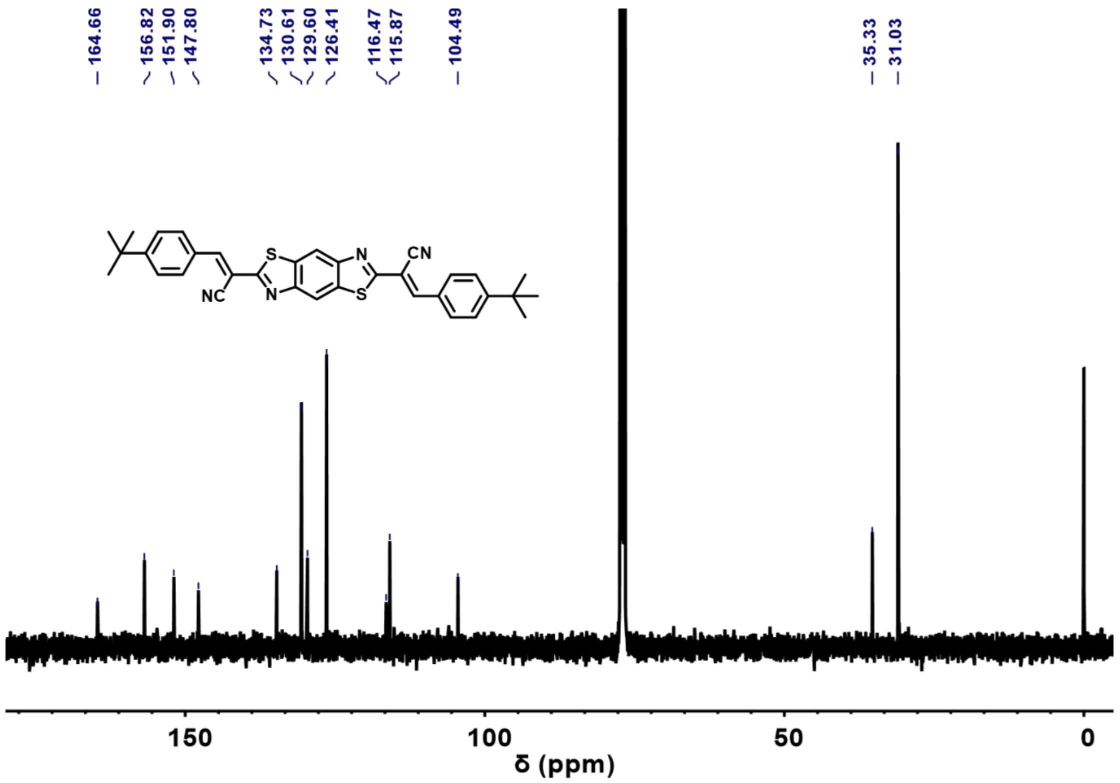

Supplementary Figure 9. ^{13}C NMR spectrum of model compound.

Analysis Info

Analysis Name	D:\Data\MALDI\2021\1112\W2_0_G12_000001.d	Acquisition Date	11/12/2021 6:03:15 PM
Method	MALDI_P_100-3000	Operator	
Sample Name	MURU-N-ESI	Instrument	solariX
Comment			

Acquisition Parameter

Acquisition Mode	Single MS	Acquired Scans	6	Calibration Date	Thu Nov 11 05:37:32
Polarity	Positive	No. of Cell Fills	1	Data Acquisition Size	2097152
Broadband Low Mass	202.1 m/z	No. of Laser Shots	10	Data Processing Size	4194304
Broadband High Mass	1000.0 m/z	Laser Power	18.6 lp	Apodization	Sine-Bell Multiplication
Source Accumulation	0.001 sec	Laser Shot Frequency	0.020 sec		
Ion Accumulation Time	0.100 sec				

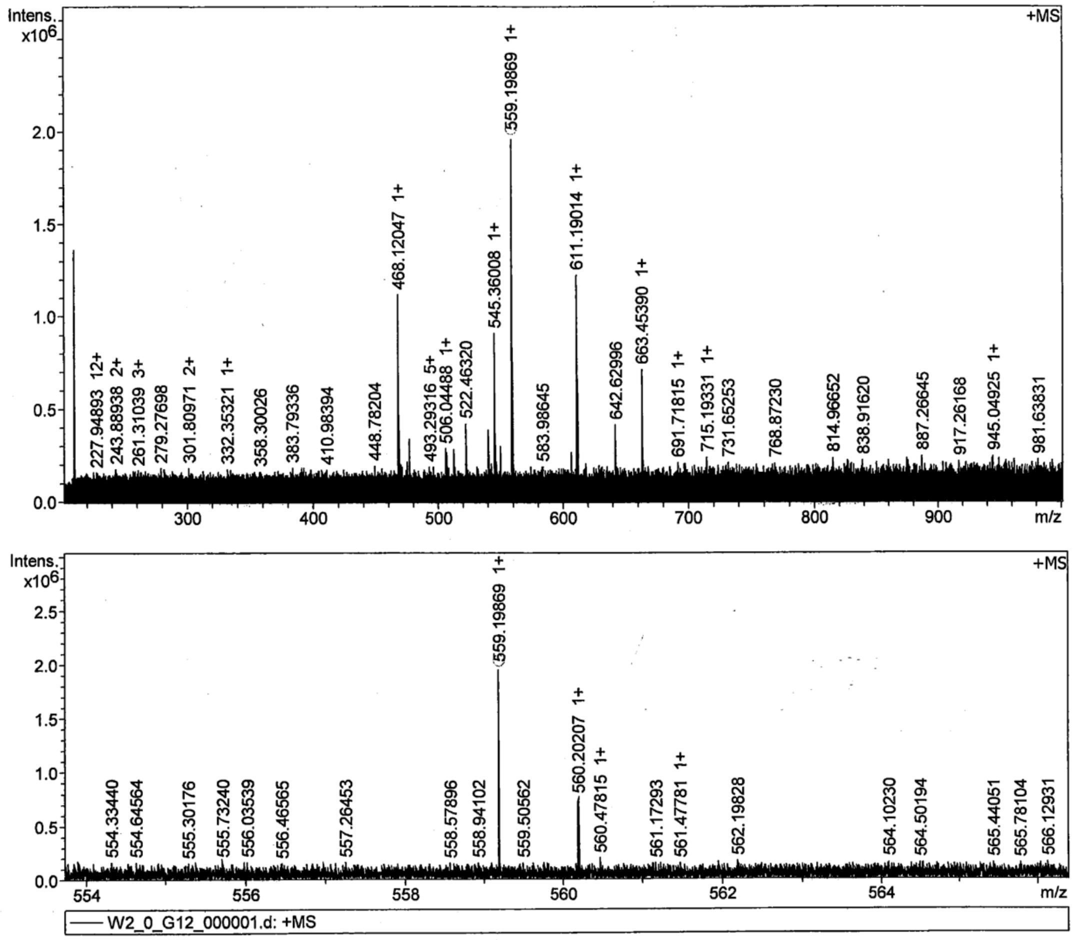

Meas. m/z	#	Ion Formula	Score	m/z	err [ppm]	Mean err [ppm]	mSigma	rdb	e⁻ Conf	N-Rule
559.198694	1	C34H31N4S2	100.00	559.198465	0.4	-0.4	78.1	21.5	even	ok

Supplementary Figure 10. HR-MS spectrum of model compound.
